# Case report: Primary pulmonary low grade fibromyxoid sarcoma progressing to dedifferentiation: probably due to *TP53* driver mutation

**DOI:** 10.3389/fonc.2024.1329264

**Published:** 2024-03-01

**Authors:** Jiawen Zhang, Haisheng Fang, Xiaomei Zhu, Chenchen Yao, Qinhe Fan, Qixing Gong

**Affiliations:** ^1^ Department of Pathology, The First Affiliated Hospital with Nanjing Medical University, Nanjing, China; ^2^ Department of Radiology, The First Affiliated Hospital with Nanjing Medical University, Nanjing, China; ^3^ Department of Pathology, Women's and Children’s Hospital Affiliated to Xiamen University (Xiamen Maternal and Child Health Care Hospital), Xiamen, China

**Keywords:** LGFMS, lung, dedifferentiated, *TP53*, next-generation sequencing

## Abstract

Low Grade Fibromyxoid Sarcoma (LGFMS), a rare entity characterized by bland histologic features, typically affects deep soft tissues of the trunk and lower extremities. Rare cases have been reported arising from the viscera and few demonstrating morphology of high-grade dedifferentiation. Here we report a 39-year-old Chinese woman presenting with primary lung LGFMS, which metastasized to the pancreas five years after diagnosis and then relapsed ten years later as a mediastinum mass. Microscopically, the lung and pancreatic lumps shared similar classical features of LGFMS, composed of bland spindle-shaped cells with low mitotic activity. However, the mediastinal mass had dedifferentiated morphology of dense sheets of round and epithelioid cells with high degree of nuclear pleomorphism and brisk mitosis. Molecular studies showed both classical and dedifferentiated areas had FUS::CREB3L2 rearrangement. However, the mediastinal dedifferentiated area presented with extra H193Y mutation of the *TP53*. Moreover, the mediastinal tumor displayed a strong and diffuse pattern of p53 expression immunohistochemically, but the primary lung and secondary pancreatic masses did not. Thus, we diagnosed the mediastinal mass as dedifferentiated LGFMS and proposed that *TP53* mutation was probably the driver gene alteration in the process, which, to the best of our knowledge, has not been reported in the existing literature.

## Introduction

Low Grade Fibromyxoid Sarcoma (LGFMS) is a low-grade malignant fibroblastic neoplasm predominantly arising from the deep soft tissues of the trunk and lower extremities among young to middle-aged adults. It’s characterized by bland spindle-shaped cells with mild nuclear pleomorphism in a fibrous and myxoid background. MUC4 is often expressed in LGFMS (1). Furthermore, LGFMS mostly harbors FUS::CREB3L1/2 gene fusions, and EWSR1::CREB3L1 gene fusion has also been reported (2). However, LGFMS can be diagnostically challenging in unusual locations with advanced morphological features. We here report a 39-year-old woman with LGFMS originating from the lungs, metastasized to the pancreas and then relapsed as a mediastinum mass, showing dedifferentiated morphology with over ten years of follow-up, and carrying FUS::CREB3L2 gene fusion and *TP53* mutation. To the best of our knowledge, this is the first published case of LGFMS harboring the mutation of *TP53*.

## Case report

**Figure 1:**
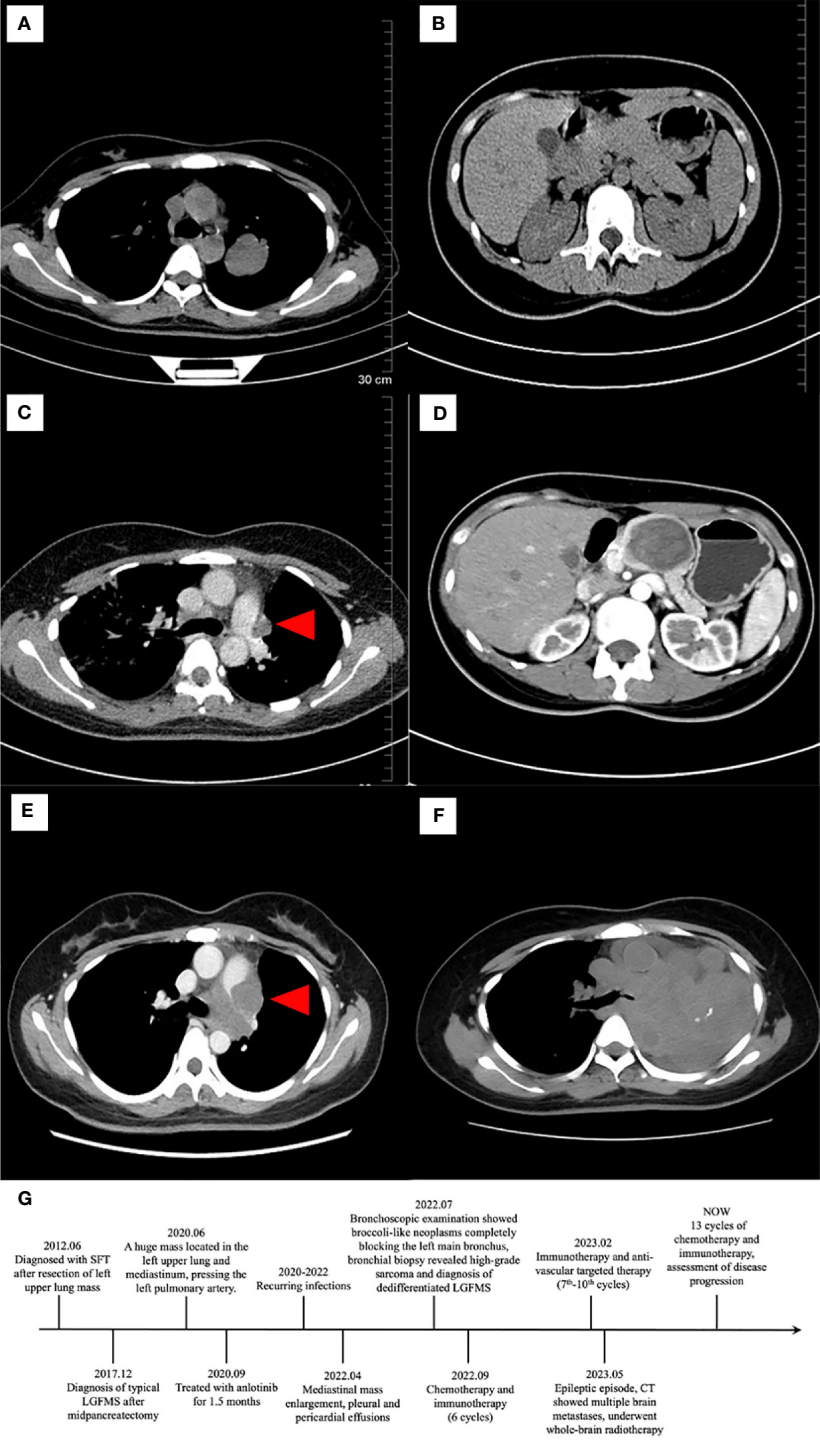
Computed tomography (CT) of lung (left) and pancreas (right). In 2012, CT showed a mass in left upper lobe **(A)**. No lesion was seen in the pancreas **(B)**. In 2016, CT found a small rise at the left edge of the main pulmonary artery (**(C)**, red arrowhead). Then in 2017, CT revealed a low-density mass measuring 60mm×50mm×30 mm in the body of pancreas, the mass being mildly enhanced and homogeneous, the pancreatic duct dilatated, and the splenic artery slightly compressed **(D)**. In 2020, there was a tumor in the left middle and upper mediastinal mass measuring 70mm×56mm×53mm, pressing the left pulmonary artery (**(E)**, red arrowhead). In 2022, on the left side of the mediastinum, a mass-like soft tissue mass was seen, with a larger size than the lesion in 2020 **(F)**. The timeline of the patient**’**s clinical history is presented **(G)**.

A 39-year-old woman was referred to our hospital due to a diagnostic dilemma concerning the left mediastinal occupancy. Her medical history revealed an admission to another hospital in 2012 for non-inducible hemoptysis. A computed tomography (CT) scan at the time revealed a mass in the left upper lobe (**Figure 1A**), initially diagnosed as a solitary fibrous tumor (SFT). No pancreatic lesion was observed then (**Figure 1B**). One year after the pneumonectomy, a follow-up CT (**Figure 1C**) scan showed a small shadow near the main pulmonary artery, considered to be a postoperative change, without further treatment. In 2017, an abdominal CT scan (**Figure 1D**) uncovered a well-demarcated pancreatic tumor (60mm×50mm×30mm), leading to middle segment pancreatectomy and LGFMS diagnosis. In 2020, a CT (**Figure 1E**) scan showed a mass (70mm×56mm×53mm) in the left upper lobe and middle mediastinum, pressing on the left pulmonary artery. As surgical treatment was no longer an option, the patient was treated with anlotinib for 1.5 months, discontinued because of recurring infections. In 2022, she was admitted to another hospital for shortness of breath. A chest CT scan (**Figure 1F**) revealed a larger mass occupying the left thoracic cavity, with pleural and pericardial effusions. Bronchoscopic examination reported broccoli-like neoplasms completely blocking the left main bronchus. A small biopsy was made and concurrent bronchial stent placement was performed. There was no recurrence of pancreatic mass then. After the diagnosis, the patient started combined chemotherapy and immunotherapy in early September 2022. The specific plan was doxorubicin (60mg d1), ifosphosate (2g d1-d3) and sintilimab (200mg d1). After the first cycle of treatment, the patient developed myelosuppression (Grade 3). After another cycle of treatment with this regimen, the patient developed nauseating pericardial effusion. As a result, the treatment was adjusted to Liposu (210mg d1), carboplatin (500mg d1) and sintilimab (200mg d1) for the third to sixth cycles of treatment, during which the patient’s condition was evaluated as stable disease (SD). In February 2023, the plan was changed to immunotherapy and anti-vascular targeted therapy with sugalimab (1200mg d1) and bevacizumab (400mg d1), the 7th to 10th cycles of treatment were performed, progressive disease (PD) was assessed during this period. In May 2023, the patient experienced a sudden epileptic episode. CT showed multiple abnormal signals in both cerebral hemispheres and the right cerebellar hemisphere. Multiple brain metastases were considered, and the patient underwent whole-brain radiotherapy (CTV-haima 30 Gy/12f). Subsequently, treatment with sugalimab (1200mg d1), bevacizumab (400mg d1) and etoposide (200mg d1-d5) were continued for 11th-13th cycles. Current assessment of disease progression. The timeline of the patient’s clinical history is presented (**Figure 1G**).

**Figure 2:**
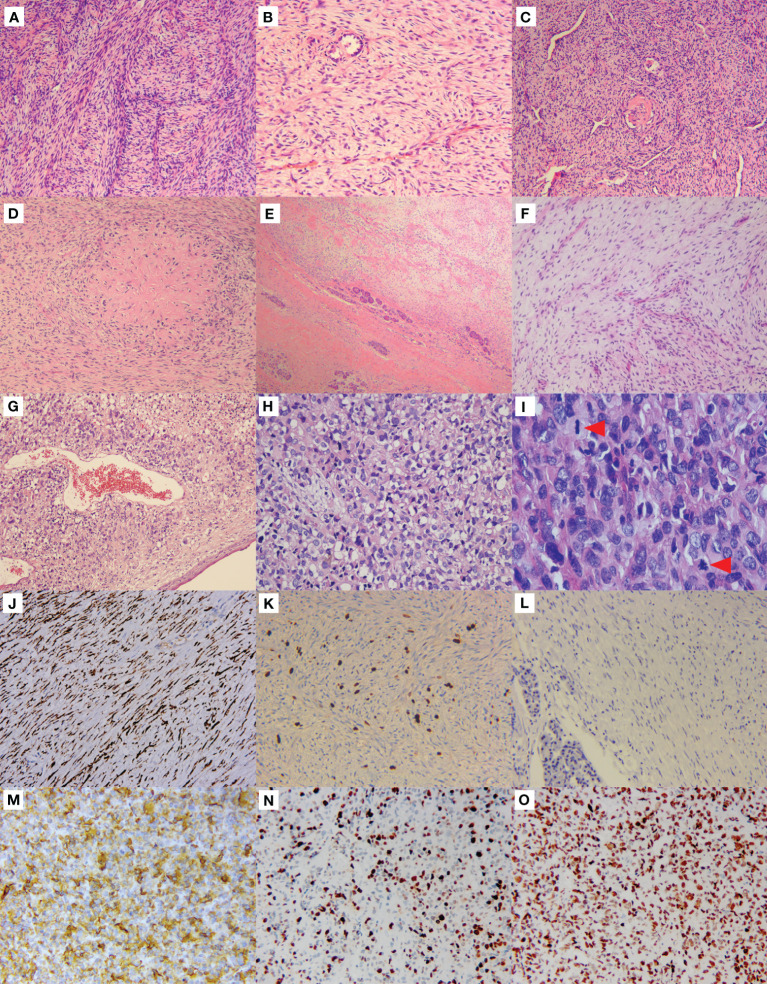
Morphological features and immunophenotype of LGFMS, a primary, metastasized, and dedifferentiated tumor. Primary lung tumor **(A–D)**. Hematoxylin & eosin (HE) staining in the low-power view showed spindle cells were densely composed and arranged in a fascicular manner **(A)**. Spindle to stellate cells were sparsely distributed in the fibrous to myxoid background, with the surrounding alveolar epithelial cells entrapped in the tumor **(B)**. Curvilinear and branching capillary-sized blood vessels were observed, some of which were dilatated as staghorn vessels **(C)**. Giant collagen rosettes characterized by a central zone of eosinophilic collagen surrounded by spindle to oval tumor cells **(D)**. Metastatic pancreatic tumor **(E, F)**. Pancreatic tissues were limitedly involved in the tumor cells **(E)**. Bland spindle cells sparsely distributed in a mucus background with thin-walled branching vessels **(F)**. Dedifferentiated mediastinal tumor **(G–I)**. Anaplastic cells were present beneath the squamous metaplasia bronchial surface epithelium **(G)**. The tumor cells were round, polygonal to epithelioid with high degree of nuclear pleomorphism and abu4ndant eosinophilic cytoplasm, and some cells showed vacuolated changes in cytoplasm **(H)**. Mitosis was active (I, red arrowheads), and pathological mitosis could be seen (**(I)**, the red arrowhead below). Immunohistochemistry **(J–O)**. The pancreatic tumor cells strongly expressed MUC4 **(J)**. The Ki-67 proliferation index was about 5%+ **(K)**, but negative for p53 **(L)**. The mediastinal tumor cells were strongly positive for MUC4 **(M)** as well. The Ki-67 proliferation index was averagely 50% **(N)**. The p53 staining was strongly and diffusely expressed **(O)**.

Histologically, the left lung mass was composed of bland spindle-shaped cells with a fibrous stroma and thin-walled blood vessels in the background (**Figure 2A**). In some areas, the spindle cells were densely composed and arranged in fascicular, whorled or random manner. While in other areas, cells with a spindle to stellate configuration were randomly distributed within a fibrous to myxoid background. Although the tumor cells appeared mild and amicable, the surrounding bronchial and alveolar epithelia were observed entrapped in the tumor (**Figure 2B**). Upon careful observation, a network of curvilinear and branching capillary-sized blood vessels was observed in both cellular and the myxoid areas. Some vessels were slender, while others were dilatated as staghorn vessels (**Figure 2C**). At high magnification, the tumor cells displayed mild nuclear pleomorphism, finely clumped chromatin, pale eosinophilic cytoplasm, and few mitotic figures. Focally, epithelioid cells were present, and a giant collagen rosette was witnessed (**Figure 2D**). There was no necrosis, and the average number of mitotic figures was approximately 1-2/10 high power fields (HPFs).

The pancreatic tumor shared similar microscopic features to the lung tumor, displaying low aggressiveness as involvement of the surrounding pancreatic tissues was limited (**Figure 2E**), but the spindle tumor cells were more sparsely distributed within a myxoid background, alongside thin-walled branching vessels (**Figure 2F**).

The biopsy of recurrent left upper lobe and mediastinal mass revealed notably aggressive morphological features. Sheets of anaplastic cells were observed beneath the squamous metaplasia bronchial surface epithelium (**Figure 2G**). Tumor cells displayed a round, polygonal to epithelioid shape with significant nuclear pleomorphism and some vacuolated cytoplasmic changes in a disordered arrangement (**Figure 2H**). Mitosis was active, with visible pathological mitotic figures (**Figure 2I**). Extensive necrosis was observed. The classical LGFMS with mild spindle tumor cells was hardly identifiable.

Immunohistochemistry of the lung and pancreatic masses showed strong MUC4 expression (**Figure 2J**), but were negative for S-100, CD117, CK, EMA, Desmin, CD34, SMA, and β-Catenin. The Ki-67 index was about 5%+ (**Figure 2K**). The mediastinal mass biopsy showed positive staining for vimentin, CD99, INI-1, MDM2, and MUC4 (**Figure 2M**), but was negative for CKp, Desmin, SMA, S-100, CD31, CD34, TTF-1, P40, STAT6, and SOX10. The Ki-67 index averaged 50% (**Figure 2N**). Additionally, the mediastinal tumor cells strongly and diffusely expressed p53 (**Figure 2O**), while the pancreatic tumor cells did not (**Figure 2L**).

**Figure 3:**
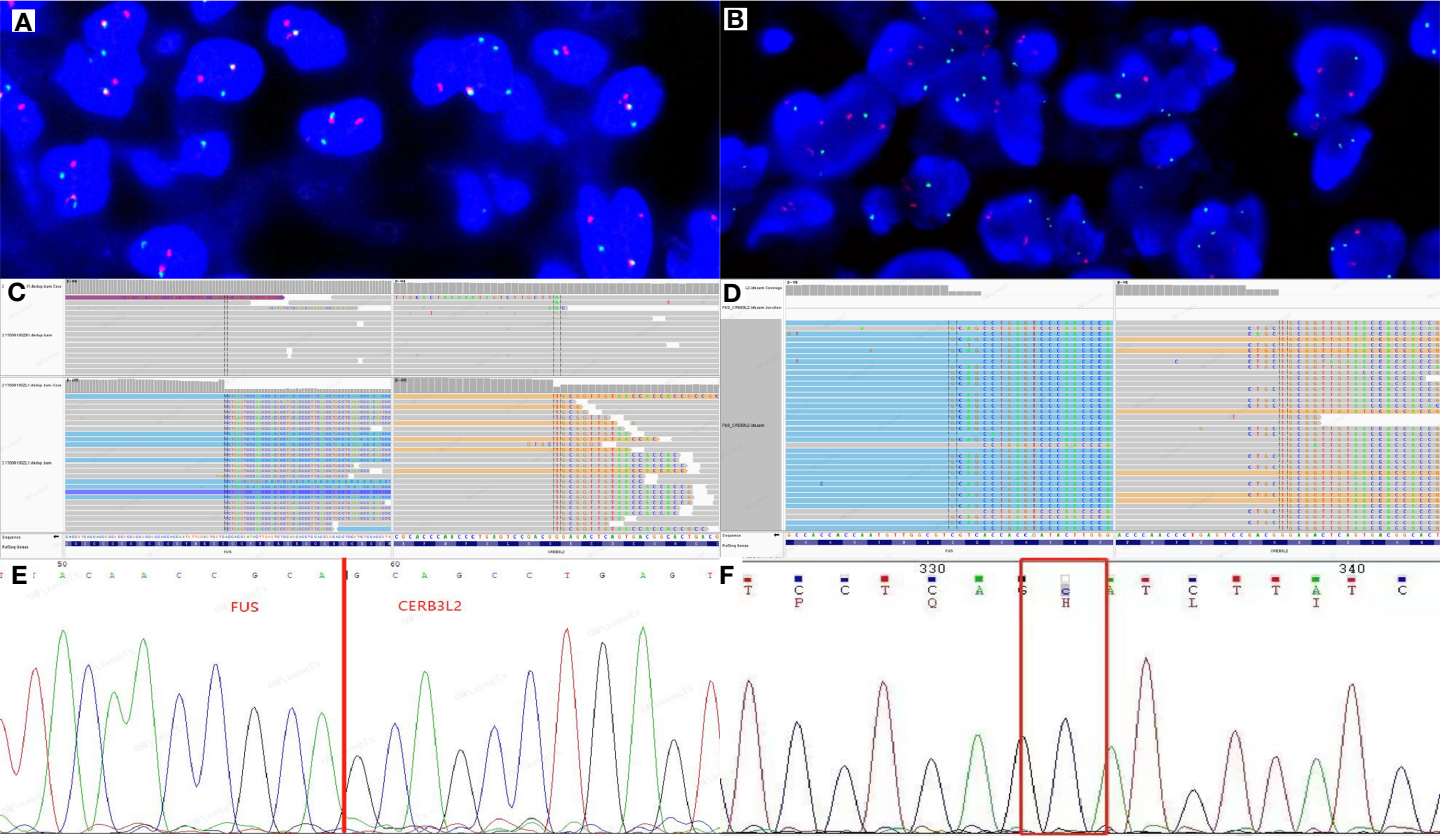
Molecular characterization of pancreatic and mediastinal tumors. Two fusions and one extra green signal were detected in more than 30% of tumor cells by FUS break-apart fluorescence *in situ* hybridization (FISH) test in pancreatic **(A)** and mediastinal **(B)** masses. Genetically, next-generation sequencing (NGS) test of the mediastinal mass revealed that FUS exon 6 was fused to CREB3L2 exon 5 **(C)** and the *TP53* had an H193Y mutation **(D)**. By direct sequencing of RT-PCR product in pancreatic tumor, chimeric transcripts were detected between FUS exon 6 and CREB3L2 exon 5 **(E)**, but *TP53* mutation was not detected **(F)**.

Dual‐color break‐apart commercial probes of FUS (Anbiping, Guangzhou, China) were used for fluorescence *in situ* hybridization (FISH) assays, showing separate signals in over 30% of pancreatic and mediastinal tumor cells (**Figures 3A, B**), indicating FUS rearrangement. Next-generation sequencing (NGS) was performed on the mediastinal mass, revealing a FUS exon 6::CREB3L2 exon 5 fusion (**Figure 3C**) and H193Y mutation of *TP53* (**Figure 3D**). Otherwise, reverse transcription polymerase chain reaction (RT-PCR) on the pancreatic tumor confirmed the presence of the same FUS exon 6::CREB3L2 exon 5 fusion as the mediastinal mass (**Figure 3E**), but *TP53* mutation was absent (**Figure 3F**). Considering these morphological and molecular findings along with the clinical history, the relapsed mediastinal tumor was diagnosed as dedifferentiated LGFMS.

## Discussion

Some low-grade soft-tissue sarcomas may undergo high-grade transformation, including dedifferentiation, namely losing their original characteristics. Dedifferentiated components can coexistxwith the original components either synchronously or heterochronously. This phenomenon has been reported in many tumors, including liposarcoma (3), chondrosarcoma, chordoma, SFT (4), gastrointestinal stromal tumor (GIST) (5), and LGFMS (6). Dedifferentiated LGFMS was first described by Evans in 1993 (7). In contrast to the classical morphology, dedifferentiated LGFMS is characterized by densely distributed epithelioid or round cells with obvious atypia. In 2006, Périgny (8) reported a case of LGFMS that dedifferentiated into a high-grade sarcoma upon recurrence, with regions resembling pleomorphic undifferentiated sarcoma and sclerosing epithelioid fibrosarcoma (SEF). In 2011, Evans reported three cases of LGFMS with high-grade transformation during relapse (6), with one case showing SEF-like changes and survived after treatment, while the other two showed high-grade round cell components and survived for only one year after dedifferentiation. More recently, Dobin (9) and Tay (10) reported molecularly confirmed cases of dedifferentiated LGFMS with FUS rearrangement validated by FISH, RT-PCR or NGS, respectively. To date, the mechanism of dedifferentiation has not been further explored. We summarize clinicopathological characteristics in **Supplementary Table 1**. The patients in this cohort exhibit a broad age range, spanning from 3 to 85 years old, with the majority of middle-aged individuals. Predominantly, the affected areas involve deep tissues of the limbs and trunk, with one case located in the retroperitoneum. Histological morphology and molecular characteristics have been described above. Surgical resection was performed in all 6 cases, with additional treatments including chemotherapy, radiotherapy, and immunotherapy in some instances. Overall survival (OS) varied from 17 months to 31.5 years, with 5 cases experiencing relapse post-surgery. Remarkably, one case remained disease-free for 17 months following surgery.

In our case, the dedifferentiated morphological features of the mediastinum tumor and misdiagnosis of the primary pulmonary LGFMS as SFT in her medical history posed diagnostic dilemmas. Initially, the mediastinal mass did not express any particular diagnostic antibodies except for MUC4, which was once considered a marker for LGFMS and SEF. However, MUC4 can also be positively expressed in synovial sarcomas, ossifying fibromyxoid tumors, and EWSR1::CREB family rearranged tumors as recently reported (11). Considering the high-grade morphology of the mediastinal tumor, the diagnosis needed more backups, although there was a history of pancreatic LGFMS. Therefore, we borrowed and reviewed the slides of the lung mass. We witnessed closed or dilated thin-walled vessels, spindle cells in fibromyxoid background, and giant collagen rosettes, which indicating the diagnosis of pulmonary LGFMS. Meanwhile, the NGS test identified FUS::CREB3L2 fusion in the mediastinal tumor. Hence, we considered the diagnosis of primary pulmonary LGFMS with pancreatic metastasis and recurrence at the original site with dedifferentiated changes. To date, there are only 4 cases of LGFMS originating from the lungs in the available English literature (12–15). The initial misdiagnosis of SFT likely stemmed from the rarity of pulmonary LGFMS and the presence of dilated staghorn vessels, which are often considered a diagnostic hallmark of SFT. However, as Papp (1) has pointed out, LGFMS can also exhibit staghorn vessels. Moreover, it’s essential to highlight that the pancreas can be a target organ for tumor metastasis (16). Hence, as in our case, when LGFMS occurs in atypical locations such as the lungs or pancreas, it warrants a careful distinction from other spindle cell tumors such as SFT, desmoid fibromatosis, fibrosarcoma, leiomyosarcoma, synovial sarcoma, etc. Immunohistochemical and molecular testing do help the differentiation. Moreover, when LGFMS undergoes dedifferentiation, it needs to be differentiated from various high-grade sarcomas such as round cell liposarcoma, small round cell sarcomas, undifferentiated sarcoma, etc. Accurate diagnosis relies on auxiliary detection and the relationship with the primary low-grade tumor or with reference to the clinical history.

Interestingly, recurred dedifferentiated LGFMS showed a driver mutation in *TP53* gene and p53 protein overexpression, but the pancreatic tumor did not. *TP53* plays a crucial role in cell cycle regulation (17). Previous studies have identified the positive expression of p53 in classical LGFMS (18), but there was no molecular test to confirm the presence of *TP53* mutation in either LGFMS or EFS. However, the presence of *TP53* mutations or *TP53* pathway-related gene alterations have been demonstrated in the progression and dedifferentiation of a variety of soft-tissue sarcomas, such as dedifferentiated liposarcoma, dedifferentiated SFT (4), and dedifferentiated GIST (5) for instance. Thus, we hypothesized that *TP53* mutations might similarly play a significant role in the dedifferentiation process in this case.

Clearly, LGFMS with dedifferentiated morphology has poor prognosis according to Evans’ study (6). For LGFMS, either classic or dedifferentiated, surgical resection is the main treatment thus far. When the tumor is removed incompletely, it is more prone to relapse. On the other hand, conventional chemoradiotherapy has limited efficacy for LGFMS. Our patient was still assessed as having progressive disease after 13 courses of chemotherapy and immunotherapy, and developed multiple brain metastases, indicating a poor prognosis.

Overall, we here describe the first case of primary pulmonary LGFMS undergoing dedifferentiation changes and associated with a diver mutation in the *TP53* gene. This case underscores the critical role of precise diagnosis in treatment. To ensure accurate diagnosis and comprehensive understanding of the disease, enough attention can never be overpaid to the application of molecular testing such as NGS, as well as careful inquiry or examine the clinical history.

## Data availability statement

The original contributions presented in the study are included in the article/**Supplementary Material**. Further inquiries can be directed to the corresponding authors.

## Ethics statement

The studies involving humans were approved by Ethics Committee of the First Affiliated Hospital of Nanjing Medical University (No.2022-SR-338). The studies were conducted in accordance with the local legislation and institutional requirements. Written informed consent was obtained from the participant/patient(s) for the publication of this case report.

## Author contributions

JZ: Conceptualization, Investigation, Resources, Writing – original draft, Writing – review & editing. HF: Conceptualization, Investigation, Resources, Writing – original draft, Writing – review & editing. XZ: Investigation, Resources, Writing – original draft. CY: Investigation, Resources, Writing – review & editing. QF: Project administration, Supervision, Validation, Visualization, Writing – review & editing. QG: Funding acquisition, Project administration, Supervision, Validation, Visualization, Writing – review & editing.

## References

[1] Papp S Dickson BC Chetty R. Low-grade fibromyxoid sarcoma mimicking solitary fibrous tumor: A report of two cases. Virchows Arch. (2015) 466:223–8. doi: 10.1007/s00428-014-1684-5 25416841

[2] Lau PP Lui PC Lau GT Yau DT Cheung ET Chan JK. Ewsr1-creb3l1 gene fusion: A novel alternative molecular aberration of low-grade fibromyxoid sarcoma. Am J Surg Pathol. (2013) 37:734–8. doi: 10.1097/PAS.0b013e31827560f8 23588368

[3] Dei Tos AP Doglioni C Piccinin S Maestro R Mentzel T Barbareschi M. Molecular abnormalities of the P53 pathway in dedifferentiated liposarcoma. J Pathol. (1997) 181:8–13. doi: 10.1002/(SICI)1096-9896(199701)181:1<8::AID-PATH700>3.0.CO;2-# 9071997

[4] Akaike K Kurisaki-Arakawa A Hara K Suehara Y Takagi T Mitani K. Distinct clinicopathological features of nab2-stat6 fusion gene variants in solitary fibrous tumor with emphasis on the acquisition of highly Malignant potential. Hum Pathol. (2015) 46:347–56. doi: 10.1016/j.humpath.2014.11.018 25582503

[5] Antonescu CR Romeo S Zhang L Nafa K Hornick JL Nielsen GP. Dedifferentiation in gastrointestinal stromal tumor to an anaplastic kit-negative phenotype: A diagnostic pitfall: morphologic and molecular characterization of 8 cases occurring either *de novo* or after imatinib therapy. Am J Surg Pathol. (2013) 37:385–92. doi: 10.1097/PAS.0b013e31826c1761 PMC3728887 23348204

[6] Evans HL. Low-grade fibromyxoid sarcoma: A clinicopathologic study of 33 cases with long-term follow-up. Am J Surg Pathol. (2011) 35:1450–62. doi: 10.1097/PAS.0b013e31822b3687 21921785

[7] Evans HL. Low-grade fibromyxoid sarcoma. A Report of 12 Cases. Am J Surg Pathol. (1993) 17:595–600. doi: 10.1097/00000478-199306000-00007 8333558

[8] Perigny M Dion N Couture C Lagace R. [Low grade fibromyxoid sarcoma: A clinico-pathologic analysis of 7 cases]. Ann Pathol. (2006) 26:419–25. doi: 10.1016/s0242-6498(06)70750-7 17255901

[9] Dobin SM Malone VS Lopez L Donner LR. Unusual histologic variant of a low-grade fibromyxoid sarcoma in a 3-year-old boy with complex chromosomal translocations involving 7q34, 10q11.2, and 16p11.2 and rearrangement of the fus gene. Pediatr Dev Pathol. (2013) 16:86–90. doi: 10.2350/12-07-1225-CR.1 23075075

[10] Tay TKY Kuick CH Lim TH Chang KTE Sittampalam KS. A case of low grade fibromyxoid sarcoma with dedifferentiation. Pathology. (2018) 50:348–51. doi: 10.1016/j.pathol.2017.09.022 29486962

[11] Agaimy A Stoehr R Otto M Brasen JH Pfarr N Konukiewitz B. Intra-abdominal ewsr1/fus-crem-rearranged Malignant epithelioid neoplasms: two cases of an emerging aggressive entity with emphasis on misleading immunophenotype. Virchows Arch. (2022) 480:481–6. doi: 10.1007/s00428-021-03140-3 PMC8986664 34228212

[12] Kim L Yoon YH Choi SJ Han JY Park IS Kim JM. Hyalinizing spindle cell tumor with giant rosettes arising in the lung: report of a case with fus-creb3l2 fusion transcripts. Pathol Int. (2007) 57:153–7. doi: 10.1111/j.1440-1827.2006.02073.x 17295648

[13] Magro G Fraggetta F Manusia M Mingrino A. Hyalinizing spindle cell tumor with giant rosettes: A previously undescribed lesion of the lung. Am J Surg Pathol. (1998) 22:1431–3. doi: 10.1097/00000478-199811000-00018 9808138

[14] Sargar K Kao SC Spunt SL Hawkins DS Parham DM Coffin C. Mri and ct of low-grade fibromyxoid sarcoma in children: A report from children's oncology group study arst0332. AJR Am J Roentgenol. (2015) 205:414–20. doi: 10.2214/AJR.14.13972 PMC4570741 26204295

[15] Yoshimura R Nishiya M Yanagawa N Deguchi H Tomoyasu M Kudo S. Low-grade fibromyxoid sarcoma arising from the lung: A case report. Thorac Cancer. (2021) 12:2517–20. doi: 10.1111/1759-7714.14107 PMC8447909 34374195

[16] Adsay NV Andea A Basturk O Kilinc N Nassar H Cheng JD. Secondary tumors of the pancreas: an analysis of a surgical and autopsy database and review of the literature. Virchows Arch. (2004) 444:527–35. doi: 10.1007/s00428-004-0987-3 15057558

[17] Seligson ND Stets CW Demoret BW Awasthi A Grosenbacher N Shakya R. Inhibition of histone deacetylase 2 reduces mdm2 expression and reduces tumor growth in dedifferentiated liposarcoma. Oncotarget. (2019) 10:5671–9. doi: 10.18632/oncotarget.27144 PMC6779286 31620242

[18] Oda Y Takahira T Kawaguchi K Yamamoto H Tamiya S Matsuda S. Low-grade fibromyxoid sarcoma versus low-grade myxofibrosarcoma in the extremities and trunk. A comparison of clinicopathological and immunohistochemical features. Histopathology. (2004) 45:29–38. doi: 10.1111/j.1365-2559.2004.01886.x 15228441

