# Supplementary material for: Case report: Primary pulmonary low grade fibromyxoid sarcoma progressing to dedifferentiation: probably due to TP53 driver mutation
Source: Front Oncol. 2024 Mar 1;14:1329264. doi: 10.3389/fonc.2024.1329264 (PMC10940343; doi:10.3389/fonc.2024.1329264)
Supplement: Supplementary file 1 [file Table_1.docx]

| Reference no | Age/ Gender | Site | Differentiated morphology | Molecular finding | Treatment | Outcome |
| --- | --- | --- | --- | --- | --- | --- |
| 6 | 38/M | Buttock | With sheets of moderately large, anaplastic, predominantly round cells | NA | Excision | Recurrences, metastases, DOD at 31 years |
| 6 | 26/M | Retroperitoneum | Composed of anaplastic round cells, collagen bundles are present focally, but the appearance is unlike that of sclerosing epithelioid fibrosarcoma | NA | Excision, and chemotherapy | Recurrence, DOD at 3 years |
| 6 | 28/F | Axilla-chest wall | Showed features of sclerosing epithelioid fibrosarcoma | NA | Excision, chemotherapy, and radiotherapy | Recurrences, metastases, AWD at 31.5 years |
| 8 | 31/M | Deltoid muscle | High-grade pleomorphic sarcoma, malignant fibrous histiocytoma type; similar to sclerosing epitheliod fibrosarcoma | NA | Excision, and chemotherapy | Recurrences, metastases, alive without disease after 19 months |
| 9 | 3/M | Thigh | Composed of sheets of epithelioid to slightly fusiform cells with moderate nuclear atypia, irregular nuclei and small amount of amphophilic cytoplasm | FUS rearrangement | Excision, chemotherapy, and radiotherapy | Recurrences, disease-free survival after 10 years |
| 10 | 85/F | Anterior chest | Had the typical appearance for LGFMS, which abruptly transitioned to hypercellular areas of high-grade dedifferentiation, consisting of epithelioid sheets and spindled cells in storiform arrangements | FUS-CREB3L2 fusion | Excision | No recurrence after 17 months |
| This case | 39/F | Lung | Sheets of anaplastic tumor cells displayed a round, polygonal to epithelioid shape with significant nuclear pleomorphism and some vacuolated cytoplasmic changes, mitosis was active, pathological mitotic figures and necrosis were observed | FUS rearrangement, TP53 mutation | Excision, chemotherapy, immunotherapy, and radiotherapy | Recurrences, metastases, AWD at 10 years |

Supplementary Table 1: Clinicopathological characteristics of reported differentiated LGFMS and our case

NA, not available DOD, died of disease AWD, alive with disease
